# Are aligners capable of inducing palatal bodily translation or palatal root torque of upper central incisors? A biomechanical in vitro study

**DOI:** 10.1007/s00784-023-05046-7

**Published:** 2023-05-27

**Authors:** Fayez Elkholy, Sophia Weber, Stefan Repky, Rudolf Jäger, Falko Schmidt, Bernd G. Lapatki

**Affiliations:** 1 Department of Orthodontics, Universitätsklinikum Ulm/University Hospital Ulm, Ulm, Germany; 2 Institute of Statistics, Ulm University, Helmholtzstr. 20, 89081 Ulm, Germany

**Keywords:** Aligner, Root torque, Bodily movement, Power ridge, Force, Moment

## Abstract

**Objectives:**

Previous studies have shown that aligners have limited ability to control root movements. The purpose of this study was to investigate which modification geometry and foil thickness are optimal for generating the force-moment (F/M) systems required for palatal root torque of maxillary central incisors.

**Materials and methods:**

Tooth 11 was separated from a maxillary acrylic model and connected to a movement unit via a 3D F/M sensor. Different modification geometries (crescent, capsular, double-spherical) with different depths were digitally implemented in the labio-cervical region of tooth 11 to induce an increased contact force. We evaluated the F/M systems exerted by aligners with thicknesses of 0.4–1.0 mm. F/M measurements were taken with tooth 11 in the neutral position and during palatal displacement of tooth 11 (simulating its initial clinical movement).

**Results:**

The mechanical requirements of palatal root torque are a palatally directed force (− Fy) and a palatal root torquing moment (− Mx). These requirements were reliably achieved with modification depths > 0.5 mm. The modification depth and foil thickness had a significant influence on − Fy magnitudes (linear mixed-effect models, *p* < 0.01). With the 0.75-mm aligners combined with 1.5-mm deep modifications, the palatal root torque range (palTR) started after an initial palatal crown displacement of 0.09, 0.12, and 0.12 mm for the capsular, crescent, and double-spherical modification geometries, respectively.

**Conclusions:**

A relatively early start of the palatal torque range (after a 0.1-mm palatal crown displacement) and appropriate − Fy magnitudes were achieved with 0.75-mm-thick aligners containing 1.5-mm deep capsular or crescent pressure regions. Subsequent clinical trials are required to confirm the clinical effects of these modifications.

**Clinical relevance:**

In vitro testing indicated that modified aligners are capable of generating the F/M components required for palatal root torque of upper central incisors.

## Introduction

In the last two decades, aligners have been widely used as a therapeutic tool for correcting tooth malpositions [1–3]. After shape-driven orthodontic tooth movement was introduced by Kesling in 1945 [4] and thermoforming of transparent foils was developed, aligners were used to treat mild tooth malpositions mostly in the anterior segment by tipping or derotation [5, 6]. Improvements in aligner techniques have allowed more complex tooth movements and have expanded the therapeutic indications of aligners [6–8].

Some of the most challenging movements for aligners are bodily tooth movements or root torque as they depend on controlled root movement [9–13]. To improve treatment outcomes and overcome underlying biomechanical problems during these challenging tasks, specific aligner modifications have been recommended [9, 14, 15]. These modifications were described by Sheridan et al. [16–18] and included composite bumps adhesively attached to the crowns or local aligner reinforcements made by indentations in the tooth model to intensify local force application [9, 14, 15]. These pressure areas have been integrated into the commercial Invisalign® system as power ridges™ [14, 15, 19]. A local pressure area can be pressed either directly into the aligner with a special plier or through a recess in the digital tooth model [20]. From a biomechanical perspective, these aligner modifications apply a relatively high local contact force on the tooth, thereby increasing the force vector in the direction of movement and generating the force couple required for controlled root movement [9]. However, these effects may be rather complex because the tooth makes contact with the aligner at multiple points and these contacts change during ongoing tooth movement [11, 15].

Whether aligners can induce controlled root movements for bodily tooth translation or true root torque remains controversial [5, 21]. Importantly, studies have not consistently distinguished between bodily tooth movement and crown tipping, or between incisor inclination changes caused by crown tipping and predominant root torque. In orthodontics, the term “torque” originates from treatment with multibracket appliances, because controlled root movement to change a tooth’s inclination is usually generated by torsion of the arch wire around its longitudinal axis [22–24]. Consequently, a force couple (i.e., a moment) is created in the bracket slot, which (in combination with an appropriate force component) may generate root torque [22–24]. It has to be noted that, independent of the appliance used, true root torque is a predominant root movement, while the center of the tooth crown remains more or less in its original vestibulo-oral position. In contrast, dominating crown movements are related to controlled or uncontrolled tooth tipping [25].

This study addresses the following three questions on aligner treatment: (1) can aligners induce bodily movement or root torque of incisors in a labio-palatal direction? (2) Do aligner modifications affect the efficacy of bodily tooth movement or torque control of incisors? And (3) which modifications are most effective at controlling root torque or translational tooth movement of incisors?

To answer these questions, we first carried out a systematic review according to the PRISMA guidelines. We searched the literature in the following electronic databases: PubMed, Science Direct, Cochrane Central Register of Controlled Clinical Trials, IEEE Explorer, and the NLM Catalog in the NCBI databases. The electronic search and an additional manual search yielded 521 articles. After excluding non-relevant articles, six studies remained for qualitative analysis, two of which were experimental in-vitro studies [9, 15] and four of which were in-vivo studies in patients (clinical trials) [12, 13, 15, 26].

Based on the results of this systematic review, we concluded the following:

- Aligners without modifications have limited potential to induce bodily movement or torque control of teeth [9, 12, 13, 26].
- Aligner modifications in the form of attachments, pressure points, or different aligner geometries might increase the efficacy of tooth translation or torque movement [9, 12–15]. However, an optimal modification geometry has not been recommended.
- Adequate staging can increase the accuracy of tooth translation and improve torque control [9, 14, 15].
- More studies, especially RCTs, are required to increase the evidence and predictability of bodily movement or torque control with aligners.

Based on these conclusions, our biomechanical in vitro study aimed to examine the effect of three different modification geometries and different aligner foil thicknesses on the 3D F/M system exerted on an upper central incisor. These pressure regions were implemented in polyethylene terephthalate glycol (PET-G) aligners in the labio-cervical crown region of tooth 11. This allowed us to systematically explore the ability of such aligners to induce a palatally directed bodily movement or even palatal root torque of upper central incisors.

## Material and methods

### Experimental setup

**Fig. 1:**
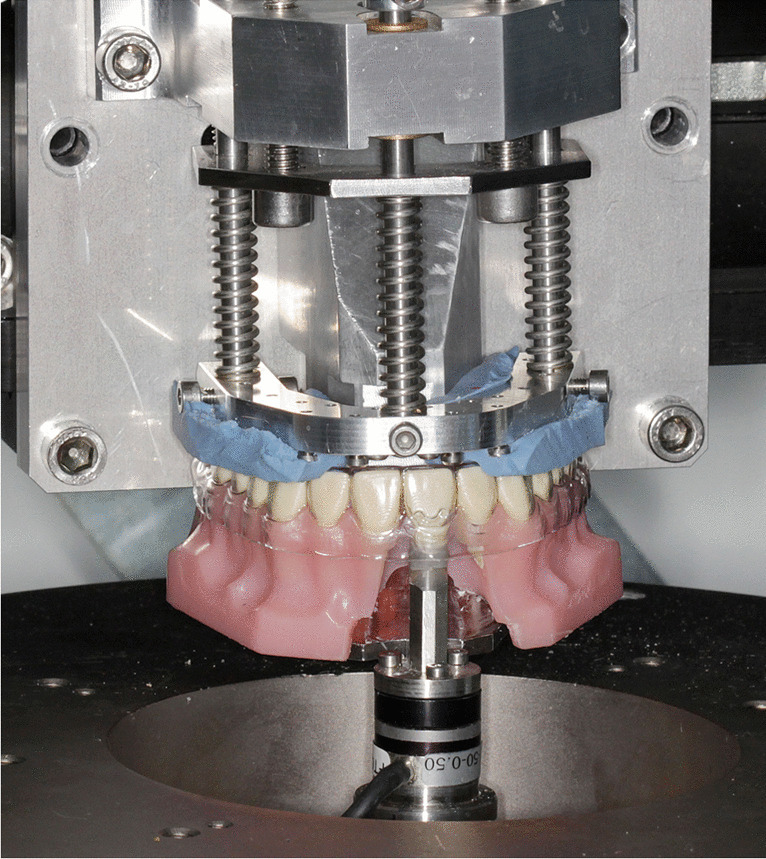
Experimental setup showing tooth 11 separated and connected to the upper ring plate of a hexapod﻿ via a six-axis load cell. A horse-shoe aluminum plate, which was lined with silicon material, was used to standardize the placement of the aligners on the acrylic maxillary arch model

The measurement setup consisted of an acrylic maxillary dentition model (Frasaco GmbH, Tettnang, Germany) with a separated right upper central incisor (tooth 11) (Fig. 1). Tooth 11 was fixed on a six-component load cell (Nano 17, ATI Industrial Automation, Apex, USA). This cell was mounted on a hexapod robot (PI M-850; Physik Instrumente GmbH & Co. KG, Karlsruhe, Germany), which allowed selective or combined 3D movements of tooth 11. To ensure secure and standardized placement of the test aligner on the acrylic model, a horseshoe-shaped aluminum plate lined with dental silicon (Silaplast Futur; Detax, Ettlingen, Germany) was used. The whole setup was enclosed in a temperature-controlled chamber maintaining a measurement temperature of 37 ± 0.5 °C.

### Test aligner modification and fabrication

**Fig. 2:**
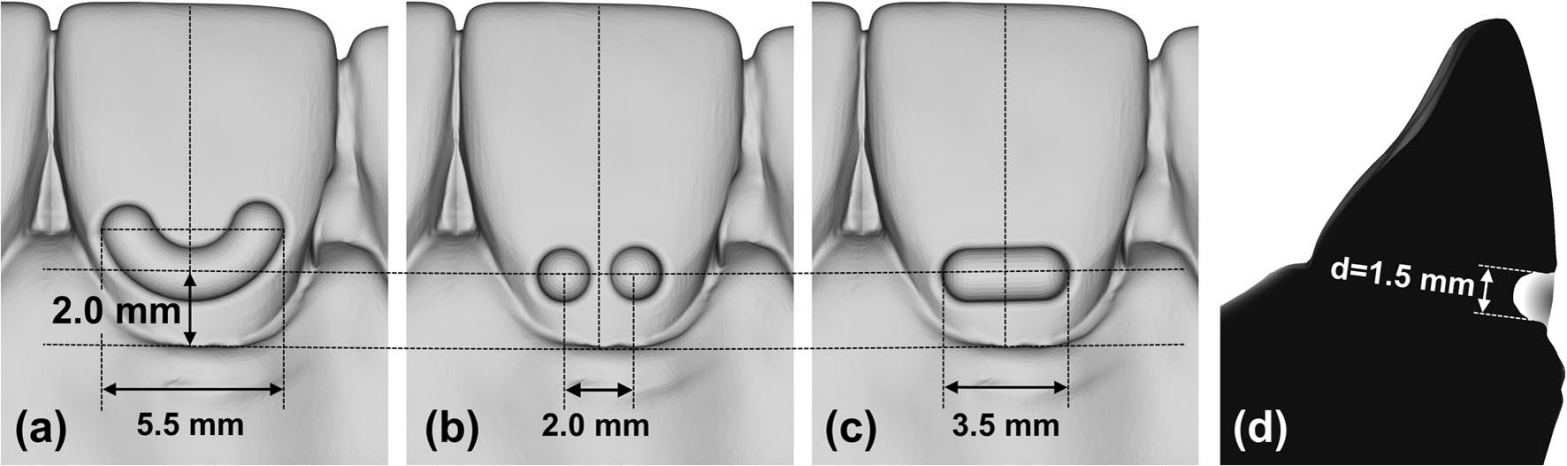
Screenshots of the digital model showing the three different modification geometries inserted 2 mm from the cervical margin of tooth 11: **a** crescent, **b** double-spherical, or **c** capsular. **d** The semicircular indentations in the model tooth had a diameter (d) of 1.5 mm. During thermoforming, the aligner foil had to follow the negative forms of these geometries

The acrylic maxillary dental arch model was digitized in a desktop scanner (d-STATION, Breuckmann, Heiligenhaus, Germany) before separating the measurement tooth. Variants of the digital model were created with indentations in the labio-cervical region of tooth 11 showing different geometries and depths using computer-aided design software (CATIA® V5-6R201, Dassault Systèmes, Vélizy Villacoublay, France). The geometries were either crescent, double-spherical, or capsular, and were embedded with their vertical center 2 mm above the gingival margin of tooth 11 (Fig. 2). This height was chosen based on pilot experiments which revealed that a minimal distance of 2 mm between the vertical attachment center and the gingival margin of the upper central incisor is required for thermoforming of the complete modification; this position also provided a sufficient distance between the dominant ﻿labial and palatal tooth-aligner contact forces leading to a sufficient moment magnitude for palatal root torque. Each shape was created with a depth of 0.5 mm, 1.5 mm, and 2.0 mm.

The nine model variants and the unmodified model were 3D-printed (Asiga Max™ IMPRIMO®, Scheu Dental GmbH, Iserlohn, Germany) using light-curing resin (IMPRIMO® LC Model, Scheu Dental GmbH, Iserlohn, Germany). Then, aligners were thermoformed on these models using PET-G foils (Duran®, Scheu Dental GmbH, Iserlohn, Germany). All test aligners were trimmed to a height of 3 mm from the gingival margin to increase their stiffness with respect to root torque application.

For the first set of measurements, we fabricated five aligners for each of the nine model variants (yielding 45 digitally modified test aligners) using a uniform foil thickness of 0.75 mm. In addition, two specific pliers (Micro Ramp Pliers, Dentsply Sirona Inc., York, PA, USA) were used to manually indent 1-mm deep double-spherical or capsular shapes into five aligners thermoformed on the unmodified maxillary dental arch model (yielding 10 manually modified test aligners). For comparison, we also tested five 0.75-mm aligners without modifications. Hence, a total of 60 (45 + 10 + 5) test aligners were examined in the first measurement set.

In the second set of measurements, 1.5-mm deep crescent, double-spherical, or capsular shapes were indented into 0.4-mm, 0.5-mm, 0.625-mm, 0.75-mm, and 1.0-mm aligners to examine the influence of foil thickness on the magnitude of the relevant F/M components. Again, five aligners were fabricated per variant, giving a total of 75 (3 × 5 × 5) test aligners.

### Measurement procedure

To simulate the intraoral milieu, each aligner was moistened with artificial saliva (Glandosane, Cell Pharm, Bad Vilbel, Germany). Then, the tested aligner was positioned on the model with tooth 11 in the neutral position.

**Fig. 3:**
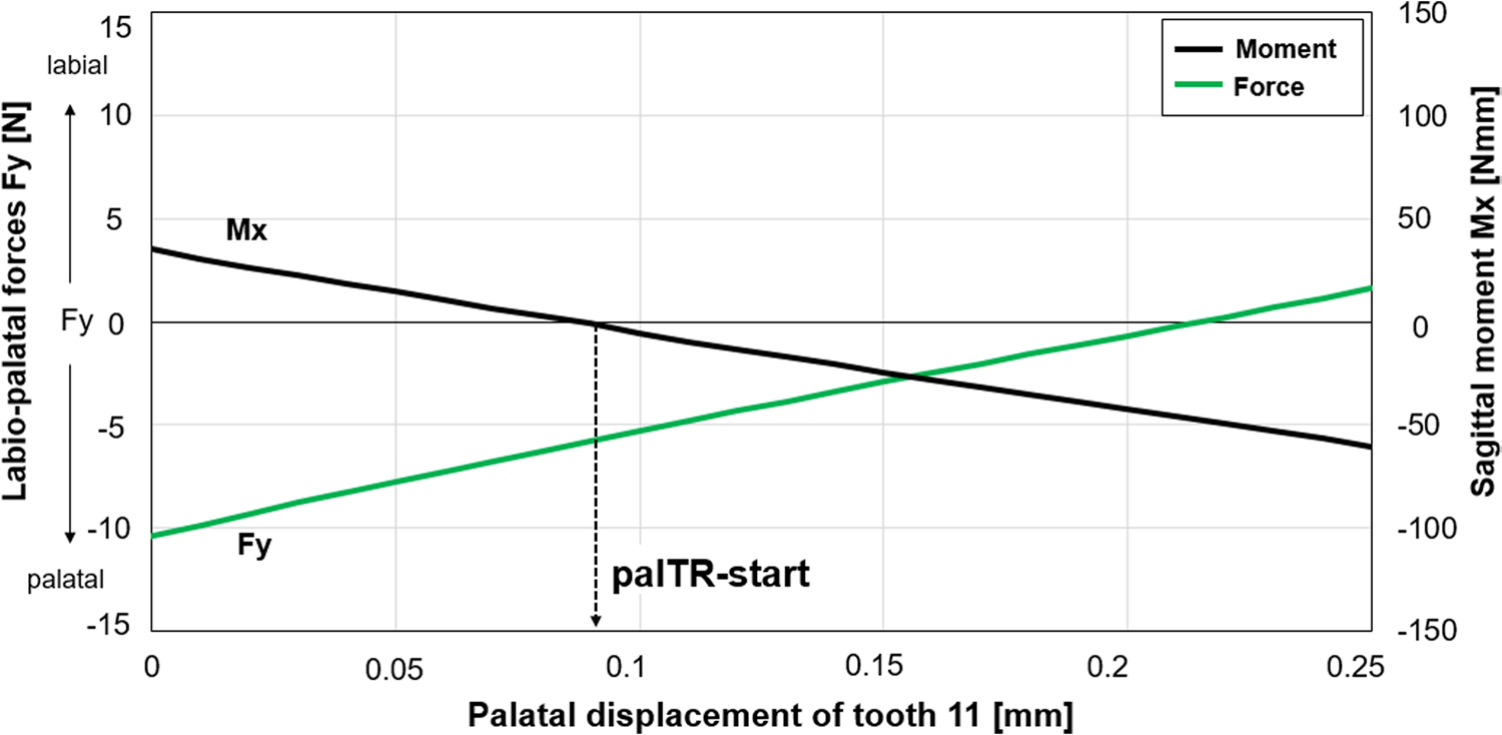
Labio-palatal force component (Fy, green curve) and the moment (Mx, black curve) in the sagittal plane measured during the simulated palatal displacement of tooth 11 after seating a 0.75-mm aligner with 1.5-mm deep capsular modification on the maxillary model. The black arrow marks the start of the effective palatal torque range (palTR-start)

Pilot measurements with modified aligners revealed a relatively high negative (i.e., palatally directed) force (Fy) in the neutral position of tooth 11 (Fig. 3) that would induce crown movement in the corresponding direction. Hence, the test procedure included a bodily translation of the measurement tooth in the palatal direction to simulate this initial tooth movement. Palatal translation of tooth 11 was performed in 0.01-mm steps to a maximum displacement of 0.25 mm to examine the effect of the corresponding tooth-aligner contact change on the F/M system exerted on this tooth. Correspondingly, the 3D F/M components were registered after each movement step.

We collected three measurements from each of the five test aligners per variant. All measurements were collected at 37 °C.

### Data analysis

Data were analyzed using Matlab algorithms (The Math Works Inc., Natick, USA) programed in-house.

For this study, we considered the labio-palatal force component (Fy) and the moment in the sagittal plane (Mx) as relevant F/M components. Measured F/M components were transformed to the center of resistance (Cr) of tooth 11. According to the literature, the Cr is estimated to be 13.8 mm from the incisal edge along the longitudinal axis of the root of an upper central incisor [27]. Such transformation of the 3D F/M system has the advantage that individual F/M values directly indicate which 3D movements of tooth 11 would be induced. For instance, if all three moment components are zero, a tooth would only be translated in the direction of the resultant force.

In the context of in vitro studies on the F/M delivery of aligners, a high mechanical stiffness of the measurement setup and measurement tooth fixture is crucial for establishing valid results. Hence, prior to our study, we evaluated the mechanical compliance of these components for relevant loading conditions by comparing intended measurement tooth displacements (i.e., the translations performed by the hexapod) with corresponding actual (i.e., effective) displacements of the measurement tooth using a laser distance sensor (MEL-M5; Wenglor MEL GmbH, Germany). These data were used to validate a finite element model of the measurement setup and to determine the effective measurement tooth displacements for all experimental loading conditions. For further analyses, we used these corrected, i.e. the effective displacements.

We also determined the amount of (effective) experimental palatal movement of tooth 11 at which the labio-palatal moment component (Mx) crossed the zero line (Fig. 3); this displacement was considered to be the start of the palatal torque range (palTR-start), because both a palatally directed force (i.e., negative Fy) and an absent inclination change (i.e., zero Mx) or even a palatal root torquing moment (i.e., negative Mx) are required for bodily movement or palatal root torque of tooth 11, respectively. Fy magnitudes at palTR-start were also extracted.

### Statistical analysis

All statistical analyses were conducted in R (R Foundation for Statistical Computing, Vienna, Austria).

Relevant variables are presented as median values and interquartile ranges. Linear mixed-effect models were used to quantitatively assess the influence of the two influencing variables (modification depth and aligner foil thickness) on Fy and Mx magnitudes as well as the palTR-start. These models were calculated separately for the different modification geometries (leading to one model per influencing variable and modification geometry) and over all modification forms (resulting in one global model for each of the two influencing variables). All models included aligner-specific random effects adjusting for repeated testing of identical aligner variants.

Variables were also evaluated pairwise using the Wilcoxon–Mann–Whitney test (α = 0.05) with Holm–Bonferroni correction for multiple testing.

## Results

Without modifications, 0.75-mm-thick aligners showed negligible Fy and Mx magnitudes with tooth 11 in the neutral position (Table 1). Furthermore, experimental palatal translation of tooth 11 induced an increasing labially directed force (+ Fy) when modifications were absent. This showed that aligners do not meet the requirements for bodily movement of tooth 11 in the palatal direction or palatal root torque of tooth 11 without modifications.

**Table 1:** Palatally directed forces (− Fy) and palatally torquing moments (− Mx) with tooth 11 in the neutral position, as well as displacements of tooth 11 at which the palatal torque range started and Fy magnitudes measured at the start of the palatal torque range, for 0.75-mm Duran® PET-G-aligners with different digitally or manually created modification geometries (crescent, double-spherical, or capsular) with different depths﻿

Modif. method*	Modif. geometry*	Foil thickness [mm]	Modif. depth [mm]*	Tooth 11 in neutral position		Start of palatal torque range (Mx = 0 Nmm)	
				Fy (IQR*) [*N*]	Mx (IQR) [Nmm]	Displ. (IQR) [mm]	Fy (IQR) [*N*]
None	None	0.75	0	0.00 (0.05)	− 0.17 (0.30)	−	−
Digital	Crescent	0.75	0.50	− 1.05 (0.68)	0.25 (5.64)	0.01 (0.02)	− 0.76 (0.35)
			1.50	− 9.80 (1.45)	41.25 (7.57)	0.12 (0.02)	− 4.84 (0.39)
			2.00	− 11.46 (1.16)	45.30 (10.04)	0.14 (0.02)	− 5.01 (0.50)
	Double-spherical	0.75	0.50	− 2.27 (0.21)	8.34 (4.27)	0.03 (0.02)	− 0.85 (1.03)
			1.50	− 8.66 (0.41)	42.32 (3.20)	0.12 (0.01)	− 3.26 (0.29)
			2.00	− 6.61 (2.59)	12.15 (18.21)	0.06 (0.05)	− 4.69 (0.28)
	Capsular	0.75	0.50	− 1.80 (0.35)	-9.89 (8.02)	0.00 (0.04)	− 0.66 (0.30)
			1.50	− 9.54 (0.83)	35.43 (9.89)	0.09 (0.02)	− 5.18 (0.70)
			2.00	− 11.85 (1.17)	48.41 (7.12)	0.15 (0.01)	− 5.35 (0.51)
Manual	Spherical	0.75	1.00	− 9.51 (10.02)	35.86 (7.36)	0.12 (0.02)	− 4.03 (0.50)
	Capsular	0.75	1.00	− 10.69 (1.23)	46.31 (9.78)	0.14 (0.03)	− 4.28 (0.69)

^*^Modif., modification; *Displ.*, displacement; *IQR*, interquartile range

Figure 3 shows individual Fy and Mx curves for 0.75-mm-thick aligners with 1.5-mm deep capsular modifications (which are representative of all aligner variants with modifications). These modified aligners induced relatively high palatally directed forces (i.e., negative Fy magnitudes) on tooth 11 in the neutral position. The Fy magnitudes for these modified aligners were approx. − 10 N, and the positive Mx values were approx. + 35 Nmm, corresponding to a reclining effect on tooth 11 (which is actually the opposite rotational direction compared to palatal root torque). As palatal displacement of tooth 11 increased, both the positive Mx and negative Fy values decreased towards the zero line. Importantly, the Fy magnitudes were still negative (approx. − 5 N) at the displacement at which Mx curves crossed the zero line from positive to negative values. Consequently, at this displacement, F/M values begin to fulfill the requirements for bodily movement and palatal root torque of tooth 11 (i.e., the palTR-start). Mx and Fy curves showed a nearly linear interrelation with increasing palatal displacement of tooth 11, and this observation was representative for all test aligners.

**Fig. 4:**
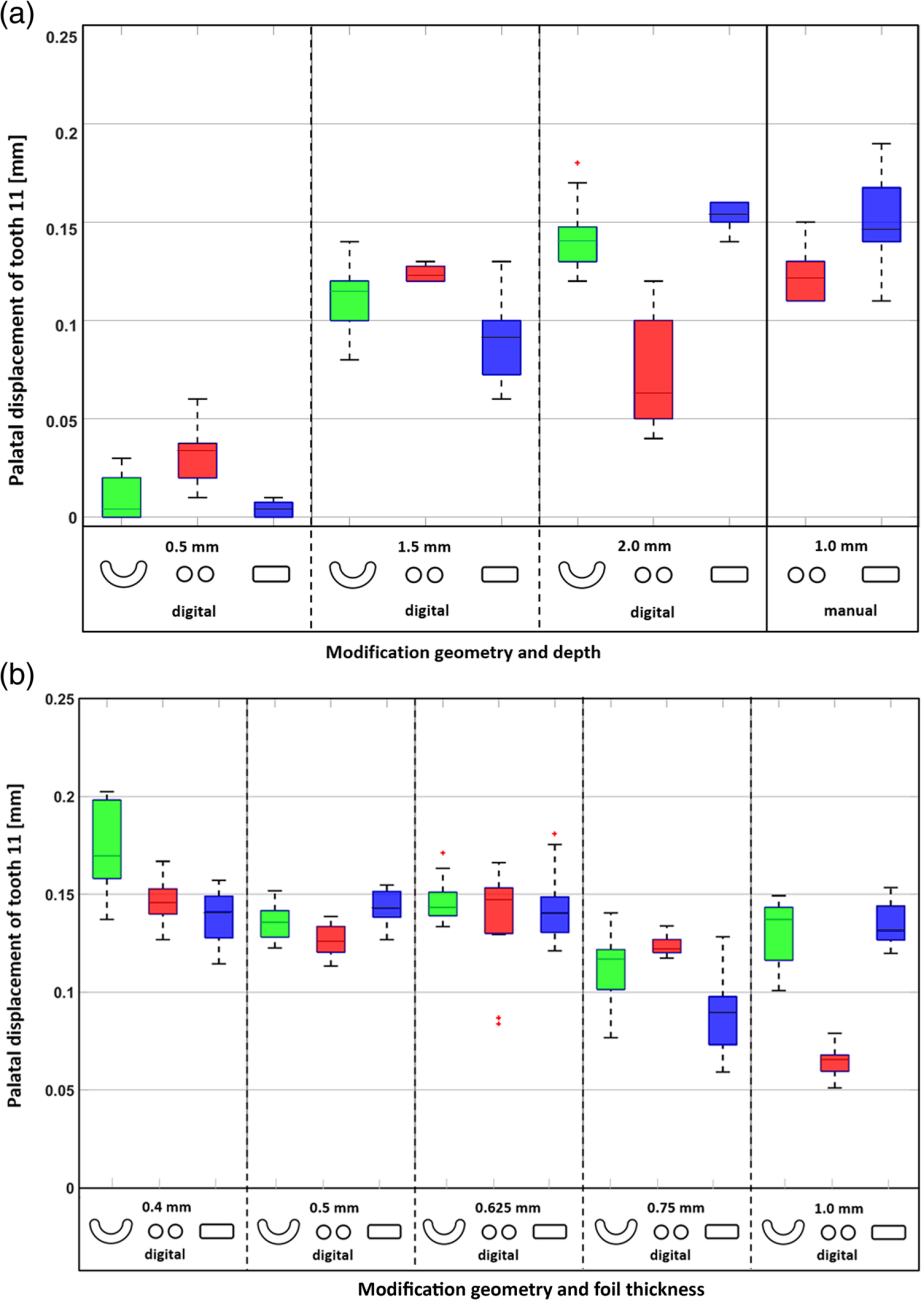
Palatal displacements at which the palatal torque range started (palTR-start). **a** palTR-start values for 0.75-mm-thick aligners with different modification geometries (crescent, double-spherical, capsular). The digital modification depths tested were 0.5 mm, 1.5 mm, and 2 mm. The manually indented modifications had either a crescent or double-spherical shape and a depth of 1 mm. **b** palTR-start values for aligners with 1.5-mm deep crescent, double-spherical, or capsular modifications according to foil thickness (0.4 to 1.0 mm)

The palTR-start displacements for all investigated modification geometries, modification depths, and aligner thicknesses are presented in Table 1 and Fig. 4a and b. All aligner variants with digitally designed or manually indented modifications achieved the palTR at a certain palatal displacement of tooth 11. Generally, we observed that both the modification depth and the aligner foil thickness significantly influenced the palTR-start values (linear mixed-effect models, *p* < 0.01).

The linear models for 0.75-mm aligners with crescent and capsular modifications revealed that a 1-mm increase in the modification depth was related to a ca. 0.10-mm increase in palTR-start displacement. Boxplots for this variable (Fig. 4a) showed that, for 0.5-mm deep modifications, the palTR started at very small displacements of ≤ 0.03 mm for all shapes. For 0.75-mm aligners with 1.5-mm deep modifications, the palTR started significantly later (Wilcoxon–Mann–Whitney tests, *p* < 0.05), at displacements between 0.09 and 0.12 mm. By increasing the digital modification depth to 2.0 mm, crescent and capsular modifications showed palTR-start displacements of 0.14 mm and 0.15 mm, respectively. In contrast, the palTR-start values for 2-mm deep double-spherical digital modifications decreased to 0.06 mm. The palTR-start displacements of 0.75-mm aligners with 1.0-mm deep manually indented double-spherical and capsular modifications were 0.12 mm and 0.14 mm, respectively.

Linear mixed-effect models of all three modifications revealed a significant interrelation between aligner thickness and palTR-start displacements; for 1.5-mm deep modifications, the palTR started 0.007 mm earlier per 0.1-mm increase in foil thickness (*p* < 0.01). However, boxplots showed that this trend and the differences between the modifications were not consistent (Fig. 4b).

**Fig. 5:**
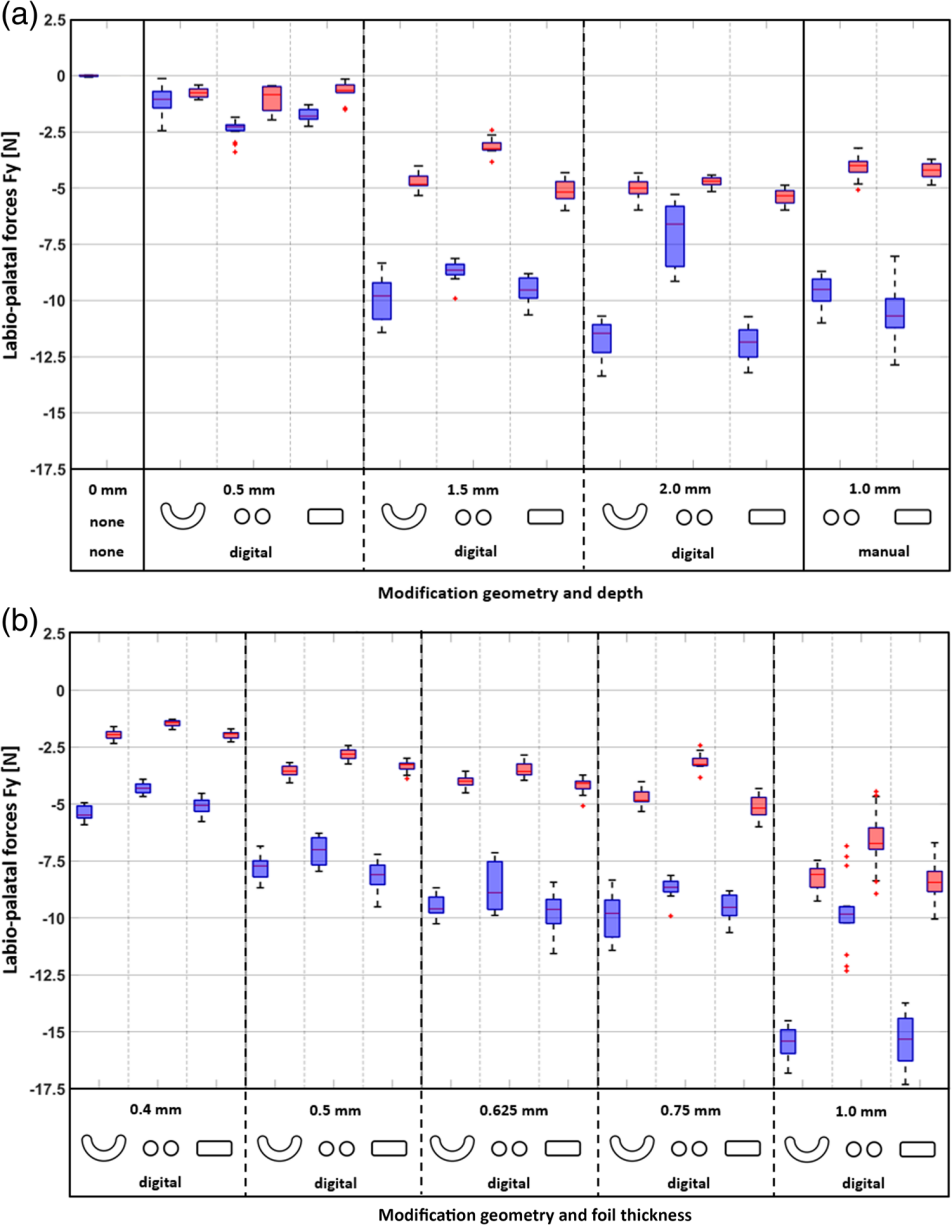
Fy magnitudes measured with tooth 11 in the neutral position (blue) and at the start of the palTR (red). **a** Fy magnitudes for 0.75-mm-thick aligners with different modification geometries (crescent, double-spherical, capsular) and modification depths (0.5 mm, 1.5 mm, 2 mm). The manually indented modifications had either a crescent or double-spherical shape and a depth of 1 mm. **b** Fy magnitudes for aligners with 1.5-mm deep crescent, double-spherical, or capsular modifications according to the foil thickness (0.4 to 1.0 mm)

The relationships between modification depth and the palatally directed forces (− Fy) are illustrated as boxplots in Fig. 5a. The − Fy magnitudes increased significantly with increasing digital modification depth with tooth 11 in the neutral position and at palTR-start (linear mixed-effect models, *p* < 0.01). Every 1-mm increase in the depth of crescent, double-spherical, and capsular modifications increased the − Fy magnitudes in the neutral position by − 7.32 N, − 3.55 N, and − 8.10 N and at palTR-start by − 2.99 N, − 2.42 N, and − 2.53 N, respectively. These estimates explain why − Fy magnitudes for ≥ 1.5-mm deep crescent and capsular modifications tended to be higher than those for double-spherical modifications (Wilcoxon–Mann–Whitney tests, *p* < 0.01 for the majority of pairwise comparisons). With tooth 11 in the neutral position, aligners with 0.5-mm deep modifications induced relatively small − Fy magnitudes with median values ranging between − 1.05 and − 2.27 N (Table 1); these values dropped to < 0.85 N once tooth 11 was displaced to the palTR-start. In comparison, aligners with ≥ 1.5-mm deep digital modifications had significantly greater median − Fy magnitudes (Wilcoxon–Mann–Whitney tests, *p* < 0.05), ranging from − 6.61 to − 11.85 N in the neutral position and from − 3.26 to − 5.35 N at the palTR-start (Table 1). In contrast to the positive correlation observed between − Fy magnitudes and the modification depth (Fig. 5a), aligners with 2.0-mm deep digital double-spherical modifications had lower − Fy magnitudes with tooth 11 in the neutral position than aligners with 1.5-mm deep double-spherical modifications did (Wilcoxon–Mann–Whitney tests, *p* < 0.01). Furthermore, an increase in the depth of digital crescent and capsular modifications from 1.5 to 2.0 mm did not significantly increase − Fy magnitudes at the palTR-start (Wilcoxon–Mann–Whitney tests, *p* > 0.05). The − Fy magnitudes of the two manual modifications were within the range of those for the digital 1.5-mm and 2.0-mm deep modifications (Fig. 5a).

The relationship between foil thickness and negative Fy magnitudes is shown in Fig. 5b and Table 2. Boxplots showed that the − Fy magnitudes increased significantly with foil thickness with tooth 11 in the neutral position and at the palTR-start (linear mixed-effect models, *p* < 0.01). This influence was greater for crescent and capsular modifications than for double-spherical modifications (Wilcoxon–Mann–Whitney tests, *p* < 0.01). Accordingly, with tooth 11 in the neutral position, the increases in − Fy magnitudes per 0.1-mm foil thickness were − 1.57 N for crescent modifications, − 1.54 N for capsular modifications, and only − 0.80 N for double-spherical modifications. At the palTR-start, the increases in − Fy magnitudes per 0.1-mm foil thickness were − 0.97 N for crescent modifications, − 1.03 N for capsular modifications, and − 0.76 N for double-spherical modifications. Accordingly, boxplots indicated higher − Fy magnitudes for aligners with crescent and capsular modifications than for aligners with double-spherical modifications (Wilcoxon–Mann–Whitney tests, *p* < 0.01); for instance, with tooth 11 in the neutral position, − Fy magnitudes for 0.75-mm aligners were − 9.80 N with crescent modifications, − 9.54 N with capsular modifications, and − 8.66 N with double-spherical modifications. These differences were even greater for 1.0-mm aligners, with − Fy magnitudes of − 15.41 N with crescent modifications, − 15.32 N with capsular modifications, and − 9.84 N with double-spherical modifications (Table 2).

**Table 2:** Palatally directed forces (− Fy) and palatally torquing moments (− Mx) with tooth 11 in the neutral position, as well as the displacements of tooth 11 at which the palatal torque range started and Fy magnitudes measured at the start of the palatal torque range, for Duran® PET-G-aligners with 1.5-mm deep crescent, double-spherical, or capsular modifications dependent on the foil thickness﻿

Modif. method*		Foil thickness [mm]	Modif. depth [mm]	Tooth 11 in neutral position		Start of palatal torque range (Mx = 0 Nmm)	
	Modif. geometry						
				Fy (IQR*) [*N*]	Mx (IQR) [Nmm]	Displ.* (IQR) [mm]	Fy (IQR) [*N*]
Digital	Crescent	0.4	1.5	− 5.49 (5.59)	29.07 (3.49)	0.17 (0.04)	− 1.95 (0.27)
		0.5		− 7.72 (0.70)	34.52 (4.25)	0.14 (0.01)	− 3.56 (0.36)
		0.625		− 9.60 (0.65)	44.04 (2.71)	0.14 (0.01)	− 4.00 (0.28)
		0.75		− 9.80 (1.45)	41.25 (7.57)	0.12 (0.02)	− 4.84 (0.39)
		1.0		− 15.41 (0.99)	50.99 (10.40)	0.14 (0.02)	− 8.09 (0.80)
	Double-spherical	0.4	1.5	− 4.30 (0.36)	25.92 (1.98)	0.15 (0.01)	− 1.42 (0.17)
		0.5		− 7.00 (1.13)	36.22 (2.49)	0.13 (0.01)	− 2.81 (0.34)
		0.625		− 8.89 (2.07)	46.98 (11.36)	0.15 (0.02)	− 3.57 (0.44)
		0.75		− 8.66 (0.41)	42.32 (3.20)	0.12 (0.01)	− 3.26 (0.29)
		1.0		− 9.84 (0.68)	23.52 (3.00)	0.07 (0.01)	− 6.73 (0.84)
	Capsular	0.4	1.5	− 5.06 (0.47)	27.19 (1.25)	0.14 (0.02)	− 1.92 (0.23)
		0.5		− 8.10 (0.76)	40.31 (4.10)	0.14 (0.01)	− 3.27 (0.25)
		0.625		− 9.63 (0.97)	43.62 (6.09)	0.14 (0.02)	− 4.09 (0.30)
		0.75		− 9.54 (0.83)	35.43 (9.89)	0.09 (0.02)	− 5.18 (0.70)
		1.0		− 15.32 (1.69)	49.09 (8.27)	0.13 (0.02)	− 8.44 (0.87)

^*^Modif., modification; *Displ.*, displacement; *IQR*, interquartile range

## Discussion

One of the main goals in patients with class II and class II division 2 malocclusions is the correction of the anterior and/or retroclined position of the maxillary frontal segment. This correction requires palatal bodily movement of maxillary incisors and/or palatal root torque. To induce palatal bodily movement, a palatally directed force (− Fy) with sufficient magnitude is required together with a moment (− Mx) to counteract the palatal tipping effect of the force. To induce palatal root torque, the magnitude of − Mx has to be further increased [9, 11, 25, 28]. A clear definition of the orthodontic term “torque” is important here. For instance, “palatal root torque” describes a pure or at least predominant root movement in the palatal direction with the tooth crown retaining its original vestibulo-oral position. This movement consists of two components: (1) a rotational component in the sagittal direction, which changes the tooth’s inclination by palatal root and labial crown movement, and (2) a palatally directed translational component, which compensates the rotational labial crown displacement. Based on these clear definitions, previous studies have shown that aligners have limited ability to induce true palatal root torque or palatal bodily movement of incisors. In this study, we have shown (in agreement with previous work [9, 11, 29]) that unmodified aligners cannot generate the F/M components needed for palatal incisor root torque or bodily movement. A potential solution—which was originally described by Sheridan et al. [16–18] and has been implemented in the Invisalign® system as power ridges™—is to introduce pressure areas into the aligner in the labio-cervical regions of corresponding incisors. These pressure areas can be created either digitally as a recess in the model or manually by indenting the aligner with pliers. In this study, we used both methods and investigated how different modification geometries, modification depths, and foil thicknesses affected the F/M system exerted on an upper central incisor.

**Fig. 6﻿:**
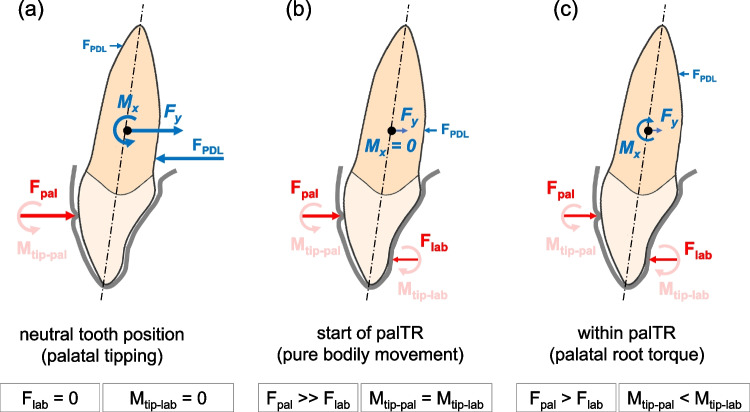
Schematic explanation of force-moment (F/M) systems exerted by the aligners with a labio-cervical pressure region by means of free body diagrams of tooth 11 for different tooth-aligner contact situations. Only the most relevant force-moment components, i. e., the labio-palatal forces and the sagittal moments, are depicted. The predominant tooth-aligner contact forces (*F*_pal_, *F*_lab_) are shown in red, and the tipping moments of these contact forces (*M*_tip-pal_, *M*_tip-lab_) are shown in light red; the blue arrows represent the resultant Fy and Mx components after transformation of *F*_pal_ and *F*_lab_ to the center of resistance of the tooth (Cr, black dot); for completion of the free body diagrams of tooth 11, the contact forces between the labial and/or palatal root surfaces and the periodontal ligament (*F*_PDL_) are also represented. **a** Contact situation and F/M components after seating the aligner in the neutral position of the tooth. **b** Situation after the initial palatal movement of the tooth to the position where the palatal torque range (palTR) started. The palatally directed contact force generated by the aligner modification (*F*_pal_) is still greater than the labially directed contact force (*F*_lab_) present at the palatal surface of the crown. Due to the greater distance of *F*_lab_ from the Cr, however, the corresponding antagonistic tipping moments of these forces (*M*_tip-pal_, *M*_tip-lab_) have equal magnitudes. Consequently, the F/M system referred to the Cr consists only of a negative total Fy component leading to pure palatal translation of tooth 11. **c** Situation within the palTR. Further palatal movement of the tooth leads to decreasing *F*_pal_ magnitudes and increasing *F*_lab_ magnitudes. Consequently, *M*_tip-pal_ values become smaller than *M*_tip-lab_ values, leading to a negative total Mx value. The latter F/M component generates root torque

The main finding of this biomechanical in vitro study was that modified aligners can generate the F/M components required for palatal bodily movement of maxillary incisors or even palatal root torque in vitro. This may be explained by strong contact at the aligner-tooth interface, which is generated by the labio-cervical pressure area of the aligner seated on the model without any setup displacement of tooth 11. The corresponding palatally directed contact force (reaching nearly − 10 N for 0.75-mm aligners with 1.5-mm deep modifications) acts cervical to the center of resistance of the tooth and generates a high positive Mx component (Fig. 6a) that tips the crown in the palatal direction (which is actually not desired). Experimental palatal movement of tooth 11 showed that negative Fy and positive Mx magnitudes decrease linearly towards the zero line (Fig. 3) during the corresponding initial clinical tooth movement. This is explained by successively reduced bending of the aligner at its labio-cervical region. The Mx values decreased because the − Fy decreased (which also reduced the palatal tipping, positive Mx moment) and because an additional moment was generated with opposite polarity (− Mx). This additional moment component was related to a successively stronger aligner-tooth contact at the palato-incisal region of tooth 11 generating a labially directed contact force; this contact occurred once the play of the tooth within the aligner was eliminated. Hence, two contact forces with opposite directions acted on different vertical levels of the tooth, building a force couple that corresponded to the additional negative Mx moment mentioned before (Fig. 6b and c). As shown in Fig. 3, the polarity of total Mx values changed after a very small initial palatal tooth displacement (i.e., palTR-start) ranging between 0.09 and 0.12 mm for 0.75-mm aligners with 1.5-mm deep modifications (Tables 1 and 2). Importantly, Fy values at the palTR-start were still negative enough (i.e., approx. − 5 N for 0.75-mm aligners with 1.5-mm deep modifications) to induce sufficient palatal translation of the tooth. Consequently, the incisor received both a sufficient palatally directed force (− Fy) and palatal root torquing moment (− Mx). It has to be noted that the reported Fy and Mx magnitudes are specific for the tested aligner types which also includes the height of the aligners in the cervical region. Future studies may investigate whether aligners with reduced cervical height have similar capabilities for generating moment magnitudes required for palatal root torque of incisors.

We also investigated which pressure-area geometries, modification depths, and foil thicknesses are optimal for generating palatal bodily translation or palatal root torque of an upper central incisor. This involved comparing the palatal displacement of the incisor at the palTR-start and the magnitude of the palatally directed force (Fy) at this displacement. Here, an important question is which force magnitude is needed for palatal bodily movement of a maxillary central incisor. Values of 0.5 ± 0.2 N have been reported as adequate [28, 30]. However, aligners start to reduce force magnitudes within a few hours to residual values of ca. 10–20% after 1 week [31]. In this respect, a force range between 2.5 and 5 N may be more appropriate. Additionally, in vivo application of aligners is confronted with a force decay due to the presence of PDL which is, however, difficult to quantify.

Our results showed that capsular and crescent modification geometries are equally able to generate high − Fy magnitudes and that both generate higher − Fy magnitudes than double-spherical modifications do. This difference was more pronounced with deeper modifications and thicker aligners, which may be explained by an incomplete thermoforming process—the smaller the negative forms of the two half-spheres and the deeper their penetration into the model, the more difficult it is for thicker foils to completely adapt to the model’s negative modification forms. Another reason might be the enclosure of air in the modification shapes during the thermoforming process, which might be more pronounced in smaller and deeper modifications. These limitations of the thermoforming process might also explain why − Fy magnitudes of 1.0-mm deep manual indentations in the thermoformed aligner were even higher than those of 1.5-mm deep digital modifications (with corresponding shape) and not smaller, as expected based on the correlation we observed between modification depth and − Fy magnitude. Accordingly, we conclude that manual modifications of aligners using pliers can also generate the F/M components required for palatal root torque.

Our results show that a modification depth of 1.5 mm is appropriate for aligners to induce palatal root torque. The − Fy magnitudes at lower depths (0.5 mm) were too small, whereas higher depths (2.0 mm) did not consistently increase the − Fy magnitudes at the palTR-start. Furthermore, 2.0-mm deep modifications increased undesired palatal tipping of the crown by around 0.05 mm until the palTR started.

Although thinner aligners (0.4 mm and 0.5 mm) fulfilled the requirements for palatal root torque, their − Fy magnitudes at the palTR-start were too small. Thicker aligners were within the appropriate range of − 2.5 to − 5 N because Fy values were − 4 N and − 5 N for 0.625-mm and 0.75-mm-thick aligners with 1.5-mm deep crescent or capsular modifications, respectively. The palTR started 0.05 mm earlier with 0.75-mm-thick aligners, so we recommend using this foil thickness for palatal root torque of upper central incisors. This recommendation, however, applies to thermoformed aligners which usually experience a considerable, non-uniform reduction of the original foil thickness during the thermoforming process [32]; hence, a direct transfer of the results to 3D-printed aligners requires caution.

Our data show that modified aligners with a sole labio-cervical pressure region and appropriate − Fy magnitudes have an offset of ca. 0.1 mm until the palTR starts. This offset may be eliminated in clinical therapy by an initial palatal movement of the crown, consisting of a dominant palatal tipping component (in the first phase) or a dominant bodily movement (closer to the palTR-start). This palatal tipping is suboptimal during correction of retroclined incisors because the root needs additional uprighting to compensate for the initial palatal tipping. Correction could be optimized by eliminating (or reducing) the initial play between the tooth to be torqued and the aligner, e.g., by adding a pressure region in the palato-incisal crown region (as in the Invisalign® system) or by suitable crown preinclination in the setup model. These alternatives and the effects of different material types are currently being investigated in follow-up experiments.

There are some limitations to this in vitro experimental study which should be considered when interpreting the quantitative results of this study. The initial movement from the neutral position of the tooth to the start of palTR was simulated as a translational movement, which is only true shortly before the palTR-start. A more realistic initial movement, considering the successive transition from palatal tipping to palatal translation (which would be very complex to realize experimentally), may result in an earlier start because the palatal tipping component would eliminate the tooth-aligner play in the palato-incisal region earlier. Another limitation is that the reported M/F magnitudes and palTR-start values were affected by the anisotropic mechanical behavior of the periodontal ligament (which could not have been simulated) [33, 34], and this influenced the initial tooth movement and contact between the tooth and aligner. Results may also have been affected by the viscoelastic aligner material behavior, which is influenced by multiple variables that are difficult to quantify [35]. Although the measurement setup was configured and optimized to achieve the maximum possible mechanical stiffness, a certain residual mechanical compliance could not be avoided, which may have influenced the palTR-start values we observed. Furthermore, our experiments simulated and investigated an isolated movement of one (measurement) tooth. In the clinical situation, however, several (if not all) teeth are simultaneously moved, which implies mutual influences on the F/M systems applied to adjacent individual teeth. This exemplifies the complexity of clinical mechanical loading of individual teeth with aligners, which is similar to complex load application of multibracket appliances. Despite these limitations, we would like to emphasize that in vitro studies are valuable for investigating fundamental research questions and for comparing different materials and modifications with maximum control. Moreover, establishing orientation values for design variables in vitro (such as the modification geometries and depths investigated here) is necessary for further testing and evaluation in patients.

## Conclusions

Modified PET-G aligners with integrated pressure regions in the labio-cervical region of an upper central incisor crown may generate sufficient palatally directed force and palatal root torque for palatal bodily movement or root torque of these teeth.

Our results suggest that 0.75-mm PET-G aligners designed with 1.5-mm deep crescent or capsular modifications achieve appropriate contact force magnitudes and a relatively early palTR-start. This seems particularly important for in-office aligner fabrication because concrete information on how aligner modifications have to be designed is either unknown or not publicly available.

Further in vitro studies are needed to minimize or eliminate the offset range before the palTR starts (e.g., by implementing additional palatal pressure points) and to compare the performance of single-layer and multilayer aligner materials. Once the design and materials have been optimized, clinical trials are needed to test the performance of these aligners.
